# How Large Is the Risk of Stagflation in the Eurozone?

**DOI:** 10.1007/s10272-022-1025-x

**Published:** 2022-02-12

**Authors:** Markus Demary, Michael Hüther

**Affiliations:** Institut der deutschen Wirtschaft Köln e.V., Konrad-Adenauer-Ufer 21, 50668 Köln, Germany

**Keywords:** E21, E31

## Abstract

The rapid recovery of demand combined with supply constraints has led to rising prices during the past months. This is evident in oil and gas markets, but also in international trade, which has been thrown out of step by bottlenecks at Asian ports. This situation creates a trade-off for the European Central Bank, because a more expansionary monetary policy cannot mitigate the supply bottlenecks and supply-side restrictions, while a more restrictive monetary policy would slow down the economic recovery. For this reason, key interest rate hikes in the eurozone are not to be expected for 2022. If the supply-side factors become persistent and wage policy tries to pass the price effects on, monetary policy will be forced to become restrictive.

As the economic recovery proceeded without disruption in the second half of 2020 following the end of the pandemic-related economic freeze in the spring, the dominant outlook was that this process would continue in 2021. The consensus forecast was correspondingly optimistic in its business cycle outlook. However, while production increased steadily in the second half of 2020 in line with the demand trend supported by fiscal policies, supply-side bottlenecks in global supply chains and logistics systems became increasingly apparent in early 2021. This is a result of COVID-19 containment measures that involved closing borders, shutting down transport systems and suspending production. Furthermore, it reflects the still manifold disruptions, especially in East Asia, caused by lockdowns based on zero-COVID strategies.

## Rising inflation – temporary or persistent?

The demand-driven recovery is a good sign following a long period of decreased demand that proceeded the debt crisis in the eurozone. Low demand has contributed to the low inflation environment that challenged the European Central Bank (ECB) in meeting its inflation target. The latest inflation numbers for Germany and the eurozone as measured by early estimates indicate that in December 2021 the costs of living have increased by 5.3% and 5.0%, respectively. The return of inflation, however, cannot be interpreted as a good sign since the recovery in aggregate demand is accompanied by supply-side frictions, which create a conflict of objectives for central banks and may create the risk of stagflation. While monetary policy can offset inflation caused by demand shocks by disincentivising aggregate demand, it has no tools to counteract supply shocks, i.e. it cannot offset supply-side frictions. While central banks can react to temporary supply shocks with a wait-and-see strategy, a more persistent supply shock might endanger the ECB's mission to meet its inflation target and force it to slow down economic growth to reduce inflationary dynamics. It is therefore important to assess the persistence of the current supply shock.

There are indications that inflation rates could indeed remain elevated for a longer period. This is due to the fact that not only have the prices of goods and services with more frequent price changes, such as food or fuels and combustibles, risen but the prices of goods and services with more infrequent price changes, such as clothing, vehicles and restaurant services, have also increased. The Federal Reserve Bank of Atlanta has created sticky-price and flexible-price consumer price indices that help to assess the persistence of inflation. This index can also be applied to the price data of other countries. In Germany, for example, goods and services with sticky prices rep-

**Markus Demary**, German Economic Institute (IW), Cologne, Germany.

**Michael Hüther**, German Economic Institute (IW), Cologne, Germany.

Figure 1

**Inflation in Germany: Sticky and flexible prices**

Percentage change from one year ago

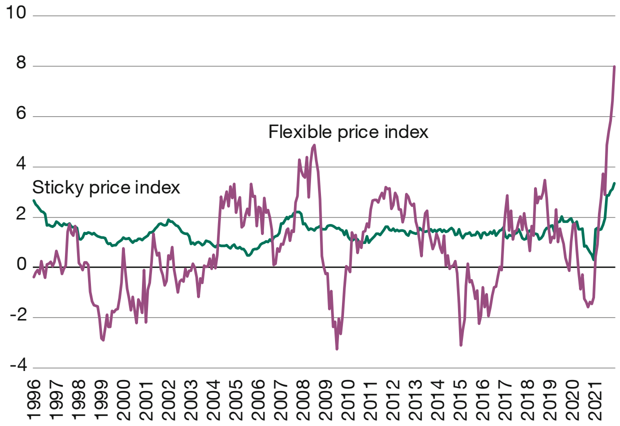

Notes: Monthly data. Sticky-price index: goods and services with less frequent price changes; flexible-price index: goods and services with frequent price changes.

Source: Bardt et al. (2021) based on German Federal Statistical Office and Federal Reserve Bank of Atlanta.

resent about 58% of the consumer price index basket. While these groups of goods have grown at a fairly stable rate of slightly above 1% for a long time, they have seen price increases of 3.5% in November 2021 (Figure 1). The rise in the sticky-price indicator highlights that there are substantial risks of persistently higher inflation rates in the future. The German economy is a heavyweight in the EU, and its inflation dynamics will to a large degree drive the inflation rate of the eurozone.

The current higher inflation rates are mainly caused by multiple one-off factors such as the reversal of the temporary value-added tax rate reduction that applied in the second half of 2020 in Germany, which will be reflected in the inflation rate in Germany for the last six months of 2021 after the reversal of the tax rate at the first of January 2021. The high savings of the household sector brought about by the lockdowns have improved the sector's balance sheet situation, increasing willingness to pay for vacations and leisure activities, which has made it easier to push through higher prices in these areas. However, the catching up of consumption has possibly only taken place in the year 2021, while part of the pandemic-related savings from the year 2020 has been invested in the capital markets rather than flowing into consumption in 2021 and 2022 (Bardt et al., 2021). This effect will also be seen in other eurozone member countries in which household savings have increased (Demary and Hüther, 2021).

The voices warning of sustained inflation are becoming louder in the face of unusually high price increases. There is concern about changing inflation expectations. Added to this are worries about a weak economic phase, which brought the term stagflation back into the media after decades. On 26 October 2021, the *Frankfurter Allgemeine Zeitung* pronounced "Germany is facing stagflation". Roubini (2021) stated early last year: "Inflation is rising in the United States and many advanced economies, and growth is slowing sharply, despite massive monetary, credit and fiscal stimulus". Stagflation is not a short-term phenomenon but rather reflects a prolonged stagnation in growth with equally persistent inflationary pressures. In the 1970s, the decade of stagflation, this resulted from a run-up to higher inflation rates, the surprise inflation caused by the oil shortage in 1973 and excessive demands on companies in the accelerated structural change under the banner of automation.

There are some factors that differentiate the situation in the past from the situation today. The wage and financial policies in the 1970s were too expansionary given the economic situation. The central banks had to build a reputation for themselves in the fight against inflation while getting accustomed to the monetary system that emerged after the collapse of Bretton Woods. The insight that a drop in prices would not solve any problems, but rather postpone them, only prevailed later. The era of accelerated globalisation and the liberalisation of international capital movements began after 1980. This was accompanied by new growth opportunities: Cost advantages and market integration in competitive markets resulted in continued price advantages for consumers.

Current stagflation risks must take permanent inflation effects into account while also analysing the growth prospects in the medium term. Decarbonisation affects both contexts because, in addition to the effects of the $CO_2$ price, it can lead to excessive demands on companies in structural change. In addition, there is the digital transformation, which is a disruptive threat to business models, and which is further increasing the pace of structural change. Ultimately, the recently strengthened protectionism and the global system conflict mean that the global division of labour does not result in any relief in price developments or in growth per se.

## The pandemic effects on international trade

Despite the manifold supply-side frictions causing delivery and performance delays in many industries and sectors and a severe setback for the automotive sector in particular, an optimistic view for overall economic development dominates 2022 and beyond. The argument put forward is that the supply bottlenecks are of a temporary nature (even if some, as in the case of semiconductors, will persist), demand is assessed globally as intact and

Figure 2
### Crude oil price and crude oil supply

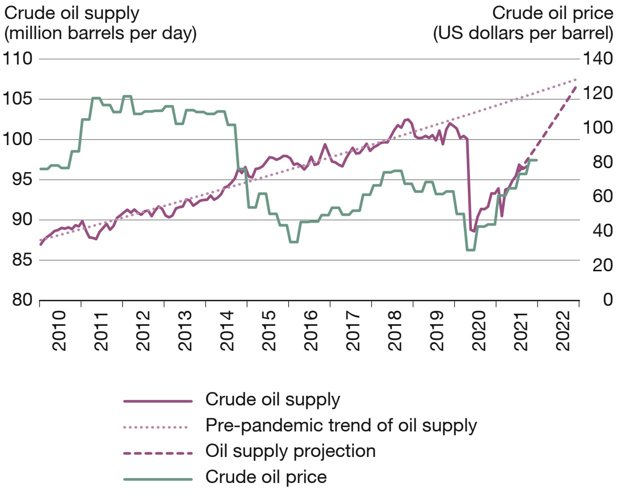

Source: Bardt et al. (2021) based on Macrobond.

Figure 3
### Temperature and European gas storage capacities

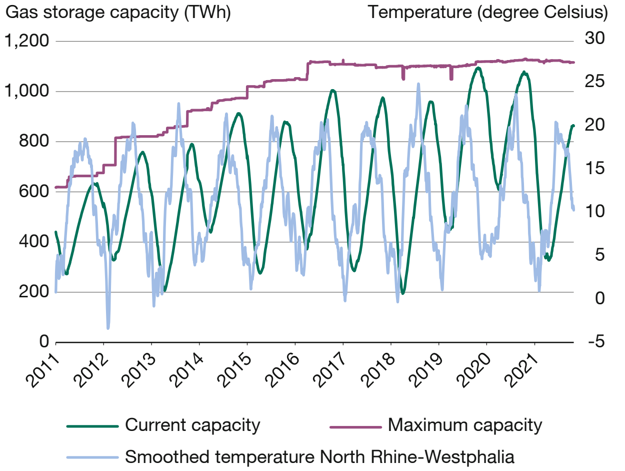

Source: Bardt et al. (2021) based on Macrobond and Gas Infrastructure Europe.

dynamic. Behind this is not least the rapid stabilisation of employment and the shortages of skilled workers coming to the fore again, coupled with the new trend that many people with medium and lower skills have sought and found other jobs during the pandemic or are only coming back from short-time work with a delay.

In addition, restrictions at Asian ports will continue, leading to high container prices and shortages of key raw materials and inputs. For example, prices of key raw materials such as alumina, aluminium, cobalt and tin have increased by more than 50% from 2020 to 2021. Due to the rise in crude oil prices, the prices of downstream products such as benzene, polypropylene and polyvinyl chloride have also increased by more than 50%. The price of kerosene has risen by 82%. In addition, urea, which is important for the automotive industry, has become 46% more expensive. Comparatively high price increases are also visible for cotton and cotton yarn and are currently reflected in the price increase for textiles. And in view of the discussion on the continued use of nuclear energy for climate-friendly power generation, the price of uranium has increased by 65% (Bardt et al., 2021).

The microchip shortage is already reflected in consumer prices. For example, the prices of computers and smartphones, having fallen on a quality-adjusted basis for many years, have now risen sharply. As these goods have made a relatively large negative contribution to inflation development in recent years, the current price increases are also making a significant contribution to inflation development. Automotive production is also affected by the chip shortage. Here, too, we are seeing significantly higher prices for the end consumer. These effects will return to normal as soon as international trade picks up again. However, friction is still expected here in the first half of this year, so price pressure will continue well into 2022 (Bardt et al., 2021).

## Energy market dynamics and inflation

There are also effects that will extend over large parts of 2022. For example, oil supply has been reduced in response to decreased mobility associated with the lockdown in the first quarter of 2021 and has not yet been able to adjust sufficiently with demand returning to normal in the summer of 2021, resulting in strong price reactions (Figure 2). In September 2021, oil supply was still 8.4% below its pre-crisis level. With the emerging positive trend in production levels, oil supply will likely return to its pre-crisis levels in February 2022. By contrast, pre-crisis oil production will not be reached again until December 2022, hence oil prices can still be expected to rise if oil production does not accelerate (Bardt et al., 2021).

Similar supply-side restrictions are evident in gas storage facilities. Storage levels are currently 22.1% below the level of the year before due to the harsh winter of 2020/21 (Figure 3). At the same time, however, temperatures started lower in November 2021 compared to the November of the previous year, resulting in a higher demand for gas for heating than the previous year. Again, higher gas prices are expected to continue throughout the winter and, if this winter is cold and gas storage is heavily depleted, will remain high next year. However, the current increases in temperature might lessen this effect. In addition, it is possible that the price of $CO_2$ has made the consumption of gas more attractive than coal, increasing the demand for gas (Bardt et al., 2021).

### The impact of the $CO_2$ price on inflation

The sharp rise in prices for energy consumption creates a trade-off for policymakers in view of their ambitious climate targets because climate policy is intended to increase prices for fossil fuels, albeit not at the current pace. Most consumers cannot change their mobility behaviour in the short term, nor can they quickly switch to a lower-emission energy source for heating. Suspending or reducing the $CO_2$ price of €25 per tonne on energy fuels and motor fuels, which Germany introduced only in 2021, would undermine the credibility of climate policy. It would therefore be more appropriate to cushion social hardship, for example by temporarily adjusting the housing allowance or higher deductions of mobility costs for commuters. In addition, consumers could be relieved of the burden of electricity costs more quickly than expected. A faster reduction or complete abolition of the German renewable energy surcharge (EEG) would also increase the incentive to switch to electricity, which is becoming an increasingly climate-friendly energy source.

In the medium term, $CO_2$ pricing will also be – and due to the goal of climate neutrality must be – reflected in consumer prices. The household sector is responsible for $CO_2$ emissions of 232.5 billion tonnes per year. Through the consumption of electricity, gas and fuels, an average German household emits around 0.2 tonnes of $CO_2$ per month. Comparatively smaller emissions of 0.04 metric tonnes, 0.03 metric tonnes and 0.04 metric tonnes per month occur via the consumption of food, vacation trips and mobility, respectively. The $CO_2$ price will increase gradually to €55 by 2025. Preuss et al. (2019) and Nöh et al. (2020) have developed a methodology for determining these effects on the cost of living. For this purpose, $CO_2$ prices are assigned to the $CO_2$ emissions of the average household. These data can then be linked to consumption data from the sample of income and consumption. Based on these data, Bardt et al. (2021) have estimated for Germany that the cost of living would increase by 0.172% from 2020 to 2021 and by 0.34% overall from 2020 to 2025 because of the $CO_2$ price, assuming it is passed on to the consumer in full. In both 2022 and 2023, the cost of living will increase by 0.03% due to the increase in the $CO_2$ price, while in 2024 and 2025 it will increase by 0.05%. Even though these are only scenario calculations, they show that the inflation rate will increase structurally, which could be a relevant constraint for monetary policy. This is because monetary policy should not actually react restrictively to these first-round effects.

The transition to a more sustainable corporate sector will not only be driven by higher $CO_2$ prices, but also by higher financing costs for those companies that rely mostly on activities not classified as sustainable in the EU taxonomy on sustainable finance. While it takes some time for companies to become green, during the transition period the financing costs for $CO_2$-intensive companies are expected to rise, thereby generating cost-push inflation in the short term.

### Labour market effects on inflation

Short-time work (*kurzarbeit*) was successfully used in Germany during the global financial crisis, and it was used at the European level during the COVID-19 crisis. The application of short-time work could have weakened the labour market effects on inflation. Demary and Hüther (2021) argue that short-time work lessens the pressures for companies to lay off workers during a recession and it therefore reduces their hiring costs during the recovery phase. The application of short-time work schemes could explain the different responses of the labour market in the US and Europe to the COVID-19 pandemic. While the unemployment rate skyrocketed in the US, it rose only mildly in Europe, increasing more slowly and to a lesser extent than during the global financial crisis and the European sovereign debt crisis.

Without the short-time work schemes, the emergence of wage-price spirals in the recovery phase would be more likely as competition among employers for new workers could push up wages, especially in those segments of the labour market where skill shortages are common. The higher wages could induce companies to increase the prices of their goods and services to compensate for the higher labour costs. As a second-round effect, higher wages give workers more financial space to increase their demand, causing higher inflation, which in turn leads workers to demand higher wages to compensate for higher prices. Up until now, the labour cost indicators do not support the emergence of a wage-price spiral, although its emergence cannot be ruled out for the months to come.

Koester and Grapow (2021) analyse the wage indexation scheme in the eurozone. They see a limited effect of wage indexation schemes on inflation, since only around 3% of private sector employees in the eurozone have their wages and minimum wages automatically indexed to inflation. Moreover, they argue that indexation schemes with a formal role for inflation developments in wage negotiations apply to around 18% of employees in the eurozone and consider mostly forward-looking inflation measures that exclude energy. The authors conclude that there is a limited contribution of wage-setting schemes triggering second-round effects, particularly when it comes to energy inflation.

This may change due to the lasting effects of an increasing $CO_2$ price on inflation and real income. The price of $CO_2$ will rise more rapidly in the coming decades in order to decrease the amount of $CO_2$; the extent of the macroeconomic impact also depends on the collateral effects along the value chain. The conversion to climate neutrality requires massive price signals to trigger the desired outcome. As the adjustment progresses, the price effects will become less important for the economy as a whole, but this is unlikely to be the case for a period of five to ten years. In order to prevent a wage-price spiral, policymakers must accept that the path to climate neutrality will have real income effects and that it is not the task of wage policy to compensate for them. What is required are policies that compensate lower-income households; otherwise, the old conflict between wage policy and monetary policy will break out again.

For the medium term, the ongoing ageing in western societies will impose a different challenge on growth and inflation. The ageing of societies reduces productivity in the medium term and therefore weakens potential growth. But productivity advances also serve to increase price competitiveness and lead to relative price reductions in competitive markets. Correspondingly, a secular productivity weakness reduces the price scope of companies. Whether wage policy will actually again be characterised by higher wage settlements (even accelerated wage growth) cannot be plausibly determined at present.

In the past, price peaks were followed by wage peaks without evident massive time-lapse effects (price-wage spiral). Scarcity premiums will increasingly create incentives to replace repetitive activities in the office with digital solutions. While the effects of automation are quite mixed in the empirical literature, it can be assumed that the effects on wages tend to be negative. It is also conceivable that the changed position of employees due to labour scarcities is not reflected in wages, but in other employment aspects such as flexible working hours. In structural change, however, employees could weight job security more heavily than higher wages, because their expected discounted sum of wage payments will increase with greater job security. Due to lower wage costs, companies have more equity to finance the investments necessary to cope with structural change, which makes jobs more secure.

## The productivity slowdown

The growth rates of labour productivity, capital intensity, total factor productivity and per capita GDP have slowed

Figure 4

**Productivity and capital intensity, in selected countries**

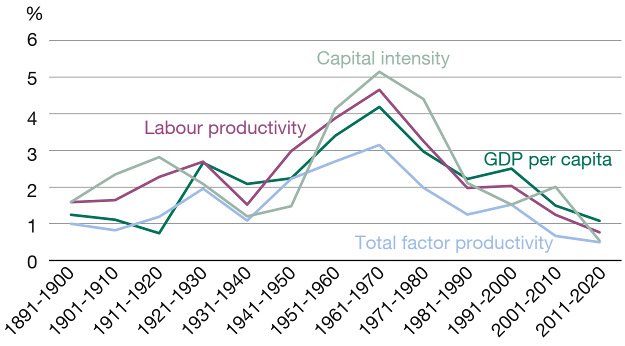

Note: The selected countries are Australia, Austria, Belguim, Canada, Switzerland, Chile, Germany, Denmark, Spain, Finland, France, the UK, Greece, Ireland, Italy, Japan, Mexico, the Netherlands, Norway, New Zealand, Portugal, Sweden, the USA.

Source: Long-Term Productivity Database, own calculations.

down since the 1960s (Figure 4). The numbers represent median value over 23 countries and ten years. The annual growth rate of GDP per capita increased by 4.2% per year in the 1960s and has slowed down to 1.1% from 2011 to 2020. During the same time span, the annual growth rate of labour productivity has slowed down from 4.7% to 0.8%, while the growth rate of total factor productivity has fallen from 3.1% to 0.5%. In addition to that, capital intensity has fallen from 5.1% to 0.5%.

Yellen (2005) argues that a slower growth in productivity has two effects on inflation. The first effect is a rise in business costs, because firms would face faster growth in unit labour cost, to which they have to respond by raising prices. Although workers will accept a lower wage growth to compensate for the slower productivity growth, she argues that there is still an adjustment period with upward pressure on inflation. The second effect is a slower growth in aggregate demand, which might offset the upward pressure of the productivity slowdown on inflation. Yellen notes that the net effect is an empirical issue.

A decline in global inflation could indicate that the second effect might have dominated the first one. While more and more central banks have implemented an inflation targeting strategy, the number of high-inflation countries has decreased, while the number of low-inflation countries has increased (Demary and Hüther, 2021). In the years before the global financial crisis and the banking and sovereign debt crisis in the eurozone, household indebtedness had increased. In the aftermath of the two financial crises, households were forced to deleverage leading to a lack in aggregate demand and thereby in subdued price growth for many years. Households have entered the COVID-19

pandemic with improved balance sheets, while the pandemic and the resulting lockdown measures have forced them to delay consumption expenditures. This in turn lead to increased saving rates and to further improvements in household balance sheets. Thus, post-pandemic household consumption could be expected to be strong because of strong household balance sheets. In combination with slow productivity growth, rising energy prices and rising labour costs, a higher inflation and low growth scenario, also known as stagflation, can be expected.

### A trade-off for central banks or a new macroeconomic policy design needed

Taking all aspects together, the risk of stagflation cannot be completely dismissed out of hand. In particular, the longer-term drivers for higher inflation and weak growth will affect not only Germany and Europe.

> There is the trend toward deglobalization and rising protectionism, the balkanization and reshoring of far-flung supply chains, the demographic aging of advanced economies and key emerging markets.…While…persistent negative supply shocks threaten to reduce potential growth, the continuation of loose monetary and fiscal policies could trigger a de-anchoring of inflation expectations. The resulting wage-price spiral would then usher in a medium-term stagflationary environment worse than the 1970s – when the debt-to-GDP ratios were lower than they are now. That is why the risk of a stagflationary debt crisis will continue to loom over the medium term. (Roubini, 2021)

While monetary policy would have to respond to an inflationary demand shock by raising interest rates, an inflationary supply shock poses a conflict of objectives for a central bank. If it wanted to combat the higher inflation rate with higher interest rates, it would further curb growth, which has already been reduced by the supply shock. On the other hand, it cannot resolve the supply constraints with lower interest rates. In doing so, it would only encourage demand and thus strengthen the inflationary dynamics. For this reason, no changes in key interest rates are to be expected this year or next, at least in the eurozone. The most likely scenario for the eurozone is a reduction in net purchases under the Pandemic Emergency Purchase Programme to zero, i.e. only expiring bonds will be replaced by new ones. However, this can only be expected if there are no liquidity bottlenecks in financial markets. Developments since the global financial crisis have shown, however, that capital markets are quite risk-averse to bad news, which may cause a sudden withdrawal of capital and thus also a sudden increase in spreads. This would be the case in light of unfavourable news about the financial stability of a country or the financial stability of a major financial institution.

Financing conditions are therefore expected to remain favourable for the time being, which should ease the economic situation. However, this will not have a positive effect against the frictions from international trade and the commodity markets. For this reason, we expect monetary policy to remain expansionary this year in conjunction with higher inflation rates. In the second half of 2022, however, inflation in the eurozone should slowly normalise towards the ECB's inflation target of 2%. Conversely, for the US, the Federal Reserve System's balance sheet is expected to shrink in the coming months. Only then will the Federal Reserve begin to raise its key interest rates. It has already had good experience with this sequence after the global financial crisis. The Fed's interest rate steps are explained by the higher demand in the US with the associated risks of overheating, resulting from the far more extensive economic stimulus programme and the infrastructure programme. The corresponding expected devaluation of the euro may have a positive impact on foreign trade, but it may also simply fizzle out due to restrictions in logistics.

The veritable risk of stagflation in the coming years calls for a macroeconomic policy design that has long appeared obsolete (Hüther, 2018). The conflict between wage policy and monetary policy finally appeared to be resolved: Falling unemployment no longer led to an increase in unit labour costs. That could change now. That is why financial policy is now being challenged from a macroeconomic perspective; it is where the key lies. It has the leeway in terms of investment policy as long as the interest rate level for long-term government bonds is below the trend of the macroeconomic dynamic.
